# Dental Hints Department

**Published:** 1907-03

**Authors:** B. H. Teague

**Affiliations:** Aiken, S. C.


### ...DENTAL HINTS DEPARTMENT...

CONDUCTED BY B. H. TEAGUE, D. D. S., AIKEN, S. C., TO WHOM ALL CONTRIBUTIONS TO THIS DEPARTMENT SHOULD BE SENT

Hemorrhage after Extraction.—In case of hemorrhage after tooth extraction, phenol sodique can be relied upon to answer every purpose.—Dental Era.

Iodoform.—There are several substitutes for iodoform which act much in the same way and have not its objectionable odor. The best of these is iodol which contains 90 per cent iodin.—H. Leonard Darrell, Dental Record.

To Prevent Expansion of Plaster in Setting.—If slacked lime is added to boiling water and the clear water decanted for use in mixing plaster of Paris, the plaster will not expand.—P. B. McCullough, International Dental Journal.

Putrescent Pulp.—On opening up a pulp-chamber in which there is a putrescent pulp giving out a most offensive odor, dip your broach in oil of turpentine and insert in canal; the odor will change instantly, most agreeably to yourself and patient.—J. E. McDonald, Dominion Dental Journal.

Removal of Trays from Muffle.—Coarse silex sprinkled over the floor of the muffle will prevent adhesion of trays, whether of clay or platinum. No attempt should be made at any time to remove the silex; most of it becomes a fixture and serves a good purpose.—Dental Office and Laboratory.

Upper Impressions.—If it is found difficult to remove an impression for full superior denture, have the patient close the lips and blow with sufficient force to distend the cheeks and the impression will drop down, no matter how tight it may have been.—R. C. Traynham, Practical Dental Journal.

Putrescent Pulp Treatment.—Excellent results can be obtained by using formalin and creosote, equal parts, to which alcohol has been added. Formalin and creosote without the alcohol will not make a clear solution; use only enough alcohol to effect this object; ten minims of alcohol is sufficient for a drachm, approximately, of the formalin and creosote.—J. P. Buckley, Dental Digest.

Grouping of Dental Drugs.—For dental purposes our drugs group themselves about as follows: Antiseptics, disinfectants and germicides; counter-irritants, caustics and escharotics; astringents, styptics and homostatics; anesthetics, sedatives and stimulants; detergents; antipyretics narcotics, purgatives; diaphoretics; emetics, and miscellaneous.—Dr. H. Clark, Dom. Journal.

Cement Bottles Should Be Kept Stoppered.—Crystallization usually results in a retarding of setting on account of water used up as water of crystallization. It can be seen that a cement liquid bottle should be kept religiously stoppered, and that the standing of liquid about the stopper must be scrupulously avoided.—Dr. W. V. B. Ames, Era.

The old rule laid down by Magnus obtains with peculiar force in the practice of dentistry, namely, that in all near work especially the illumination should neither be too strong nor too weak; it should imitate as nearly as possible diffused sunlight, and it should always shine upon the work to be done and never, either directly or by reflection, into the eyes of the operator.

Cutting Up Solder.—Solder should be used in as large pieces as can be put on the work. One or two pieces the right size for a crown will give better restlts than twenty small pieces. The reason for this is that oxide on a large piece is much less in proportion to bulk than the skin on a small piece and the weight of the large piece will break the skin easier. Again less borax is necessary, and the least borax used the better, provided you use enough.—C. C. Allen, Western Dental Journal.

Finishing Rubber Plates.—A very glossy surface can be given vulcanite work by using a dry and moderately stiff brush-wheel charged with talcum powder after having finished with pumice or emery.—F. A. Weld, Dental Review.

Polishing Vulcanite Plate.—Use aqua ammonia to mix the whiting instead of water, and the result will be quite satisfactory. After polishing, wash the plate in tepid water and finish with a clean buff wheel.—W. J. Robinson, Stomatologist.

Oral Hygiene.—To prevent fermentation of food debris and to keep the buccal cavity in a cleanly and aseptic condition, prescribe phenol sodique as a daily mouth wash. It also gives most satisfactory returns as a local application to pus pockets in pyorrhea treatment.—The Dental Era.

Inlay Adhesion.—Before adjusting an inlay the cavity should be dried and wiped with cement liquid; the inlay should be treated in the same way and the surplus liquid wiped off. This will produce a stronger adhesion when the inlay is cemented.—W. H. Upjohn, Dental Summary.

To Make Hard Plaster Modfls.—Mix the plaster of Paris with one-sixth of its volume of pulverized slaked lime. After the model has dried, place it in a 10 per cent solution of zinc sulphate until it is thoroughly saturated. Remove and dry. —Dental Era.

A Polishing Material.—To polish gold crowns, etc., I run my felt wheel over a cake of "lava soap." Very little pumice will be required, the base of this soap being probably pumice or volcanic dust. It is par excellence for the above use.—J. K. Willis, Kansas City Dental Journal.

Never haggle with a patient over a fee. Nothing is so unprofessional or demeaning. Have a just fee in accordance with the services rendered, and stick to it. The public are always suspicious of and lose respect for the man they can beat down.—Dr. S. D. Ruggles, Summary.

If the hypodermic syringe leaks around the packing, cut some washers out of base-plate gutta-percha, put in place and warm slightly over an alcohol lamp, then screw up tight.—Dr. J. A. Wells, Western Journal.

Root Canal Filling.—After pulp removal under pressure anæsthesia I always leave a small quantity of mummifying paste (zinc oxid, alum and thymol) at the extreme end of the canal and then fill with gutta-percha points dipped in a saturated solution of thymol in oil of cinnamon. The cinnamon gradually evaporates, leaving a layer of thymol crystals, lining the root canal.—Wilfred E. Griffin, British Dental Journal.

Gutta-Percha for Mounting Bridgework.—The use of gutta-percha for mounting fixed bridgework is becoming more and more general in proportion as its advantages are recognized and its manipulation mastered, the advantage offered lying mainly in the comparative ease with which the bridge may be removed in the event of necessity, and without injury to the abutments.—H. I. Goslee, Items of Interest.

On the Process of Swaging.—See that your plate crosses the die from side to side with the grain of the plate in rolling. Keep tissue paper between the plate and the die and also the counter, you will thus prevent particles of lead and zinc from being driven into the plate. Anneal three or four times, being careful to not overheat. For the last drive in swaging use no paper.—Geo. D. Sitherwood, Dental Register.

Repairing a Lower Plate.—Drill two small holes through the plate on each side of the fracture, insert binding wire through these and twist the ends together. This will bring the broken parts into correct relationship and hold them while model is being obtained.—A. W. Thornton, Dental Review.

When model is obtained put a staple, made of a brass pin, in the holes.—Editor.

Protective for Porcelain During Soldering.—When circumstances make it necessary to near-by soldering to invest crowns or bridges porcelain-face up, cover the porcelain with thin asbestos paper saturated with the investment mixture, catching the free ends in the body of the investment proper. The paper protects throughout the operation from the direct action of the flame.—Dental Office and Laboratory.

To Keep Hypo' Needles.—The best thing I can find to keep my Hypo' Dental Needle in is Glycothymoline, being alkaline it don't rust. I have a small ounce vial with cork stopper; have it most full of Glycothymoline and drop needle or needles into it. I used to use alcohol but found that it rusted the needles.—L. W. Jordan, Bingham, Maine.

Square-end Bur in a Narrow Fissure.—The worst thing I know of in the narrow fissure is the round bottom to the cavity as made with a round bur. In such a cavity your gold will roll and twist, and refuse to lie still. No wonder it is difficult. Take a bur with a square end and cut the fissures leaving square angles along the pulpal wall, and you have no need to build up between the cusps. The gold will stay where you put it. It is then comparatively easy to make perfect margins. One of the secrets of bad fillings has been the making of cavities where the gold will roll; but if you make sharp angles in your cavities you ought not to have any difficulty.—G. V. Black, Dental Review.

To Remove a Pivot.—First remove as much of the cement around the pin as possible with a small bur, being careful not to go too deep, as the bur might cut through the side of the root. Then take an inverted cone bur (No 40 to 42 S. S. W.) to the lathe wheel and grind it down until you have a long, sharp three-sided spear-pointed drill. With this follow down the side of the pin, cutting out the cement. Alternate your drilling with gently trying the pin with a small pair of pliers, and you will be surprised at the ease and rapidity with which broken pins can be removed.—Dr. C. H. Van Deventer, Cosmos.

Air in Fillings.—I may surprise some of you when I say that most of your amalgam fillings are from 12 to 16 per cent air. The same is true of your gold fillings, but with amalgam it is almost impossible to get the air out.—G. V. Black, Dental Review.

Fistula : Sinus.—It is not correct to say that an abscess has a fistulous opening. A fistula is an opening that carries a normal secretion; a fistula never carries pus. If we have an opening from an abscess carrying pus, that is not a fistula but a sinus.—A. D. Black, Dental Review.

We Are Now a Profession.—Judge Taylor in the circuit court at St. Louis, Mo., has handed down a decision in which he declares that although the practice of dentistry may formerly not have been regarded as one of the learned professions it is so regarded today and takes rank with the practice of medicine and the law. He further states that a citizen has the right to engage in any business that is not prohibited by some special law, but not so with the learned professions. The case was the State of Missouri against the National Dental Parlors.—American Dental Journal.

Oral Hygiene.—As an especially effective wash to decrease the ravages of caries, mercuric chlorid in the strength of one to twenty-five hundred forms a valuable constituent of the prophylactic dentist's armamentarium. In many mouths it will not be indicated, but in those mouths passing through the stage of extreme susceptibility it will be prescribed.—George E. Hunt, Dental Digest.

To Repair a "Morgan Matfild Mandrel."—When the pins are broken off, take a small bur and drill close to the pin through the color, then take a neuve broach, the small end to the hole, put your mandrel in a vise take a pair of pliers hold the broach and drive it light, then cut off the broach and sharpen to point. Your mandrel is as good as new.—Dentist, Holyoke, Mass.

Very good as it comes verbatim et literatum from the state in which is the Hub.—Editor.

Making Bands For Orthodontia Cases.—When making bands to retain ligatures or expansion wires, pinch the band on the side of the tooth where it is desired to have the retention. After soldering, do not cut the ends close. File or grind a slot across the ridge made by the soldered ends, sufficient to receive the ligature or wire.—R. E. Sparks, Review.

Obtunding Sensitive Dentine.—For obtunding sensitive dentine, Dr. J. Edw. Line of Rochester uses a solution of cocain on the bur while excavating the cavity. As he excavates the cavity, he runs the bur very slowly, and the solution is forced into the tubuli of the dentin, producing a very satisfactory degree of anæsthesia in advance of the bur. I have successfully tried this method a number of times.—Dental Cosmos.

Method of Inserting Crystal Gold.—How many dentists in using crystal gold have taken advantage of the ease of adaptability of some of these forms of crystal gold to a cavity by using them in connection with foil or heavy gold, placing a mass of crystal gold relatively in position, and over it a mat of foil or plate of rolled gold to facilitate the carrying of the crystal accurately to place en masse without the chopping up that is apt to occur with any form of crystal gold? It seems to me there is a saving of time and gold, and facilitating of the packing, by using alternate layers of crystal gold and foil in some form, combining in this way the virtues of foil and crystal.—W. B. V. Ames, Dental Review.

Dangers of General Anesthetics.—Two dangers must be considered: First, that of depressing a heart that is already at a disadvantage, and second, that of lessening the oxygenation of the blood, which might lead to embarrassment of the heart by distention of its chambers; for venous blood does not pass readily through the capillaries. Nitrous oxid as usually given, without oxygen, would present the latter danger, and chloroform the former, because of its marked depressant action on the heart.—Dr. E. H. Long, Cosmos.

The stability of a crown may be greatly increased by a staple made of, say No. 24 platinized wire (that used in binding magazines is all sufficient) extending well into the shell, and firmly fixed to the stump by the ends bent at a right angle and inserted into a pit drilled in each side with a groove for the wire to lay in. When the shell is cemented it holds the ends of the staple firmly in their sockets.—JOHN WARNER KEYES.

HOLLOW INLAYS.—Where I use low fusing porcelain I have gotten into the habit of laying on the inside of the matrix little pellets of gold of proper size, and placed where it would serve to help retain the filling. I am in the habit of varnishing the inlay with shellac, that there will be no adhesion of the gold to the porcelain so as to change its form. After the inlay is fused the pellets may be picked out, leaving cavities into which the cement will flow, and it gives that much of an additional hold to it. For high fusing I have used plumbago—ordinary lead pencil. It will stand the heat and I have observed no change in the shade from the use of it.—C. B. ROHLAND, Alton, Ill.

LENGTHY FUSING.—I placed a button of Consolidated body in the furnace and closed the door with the lever on No. 1 contact. Just then I was called away, and was gone for a considerable length of time, and when I came back, I took out the body, and to my surprise it was beautifully fused. I supposed then that the long run on No. 1 with the door closed had brought the temperature up to the fusing point of that body which is given as 2550 degrees Fahr. I now know my mistake for by the pyrometer I can show that under the conditions in which the furnace was working at the time it would be impossible to obtain over 2100 degrees with the door closed and the lever on No. 1, no matter how long it should be left. So I knew that porcelain fused at 2100 or less.—DR. R. M. PELTON, Review.

ACONITE AND CHLOROFORM.—Dr. Louis Jack says that after capping a pulp there is sometimes a tendency to pain in the immediate region, which is also often reflected to the ear, and a decided reaction to cold applications. In such cases, dry the gum tissue and rub away adhering mucous and apply on a pledget of cotton, for from fifteen to twenty seconds, a lotion made of one part chloroform and two parts aconite. The action of the chloroform is to accelerate the absorption of the aconite, which depresses the sensory nerves and equalizes the blood pressure at the apical region. The chloroform also acts as a counter irritant primarily with a secondary sedative reaction. Such applications have an endurance of several hours, or in some cases for one or two days.—Review.

To Tell the Need of Glasses.—There is another well recognized rule that will enable most people to decide whether they need glasses for their near work. When an individual finds that he cannot, at ten inches in front of him, read, in a dim light, "diamond" print with eoch eye separately, he ought to have glasses to assist him in his work. Nothing is to be gained by waiting, because the eye is being strained in the meantime. Of course, if one will give up near work and live an outdoor life for nine months out of the year, glasses of this description are not necessary and would be entirely superfluous, but when the dentist, the oculist, bookkeeper, stenographer, et hoc genus omne persist in working all day long for eleven months out of the year, special arrangements are generally necessary for such abuse of the eye.—Dr. C. A. Wood, Review.

To Slit and Remove a Crown.—Use a crown slitter. If you do not own one, or if you do own one that fails you, use your wedge-cutter, setting the distant angle of one blade under the edge of the gum, the other on a bit of wood on the occlusal surface of the crown. If this means fails also—and it will, quite often—drill a small hole in the crown a little distance from the grinding surface, and into this hole insert the angle of a thin chisel fixed in your automatic plugger. A few blows will slit the shell to the gum. Turning back the wings thus formed with a thin burnisher and giving the shell a pinch in a pair of hollow-nosed pliers completes the operation.—DR. W. H. ELLIS, Dom. Magazine.

HOW TO EXTRACT TEETH PAINLESSLY.—In all cases where a local anæsthetic is indicated, my method of procedure is first to have a small and very sharp hypodermic needle. Insert under the mucous membrane about one quarter of an inch from the cervix of the tooth, and inject enough of the anæsthetic to raise a little blister about the size of a small pea, remove the needle and work the blister toward the tooth with finger; this will anæsthetize the tissues around tooth sufficiently so that all following injections will be painless. Enough anæsthetic should then be injected to blanch the gums, after which lance the gums entirely away, which admits of a better grip on the tooth and prevents any injury to the gums. Take plenty of time and do the work thoroughly, and you may be assured of another patient won. —F. H. HOOD, Adrian, Mich., Summary.

PAINLESS EXTRACTION—The following is my solution of the painless extraction of teeth:

R Cocaine hyd......................grs. X.
Carbolic Acid (cryst).
Gum Camphor.
Chloral hydrate aa.... ........grs. III.
Aqua dest. q. s..................oz. IV.

Subscription—Rub camphor and chloral together until liquefied; add acid, cocaine and, lastly, water, and filter. Signature—For painless extraction. I have had it compounded a great many times at 25 cents for IV ounces. Compare the price with your $1.00 an ounce solution and compare it in an any other way, for it will bear comparison. I compound my own solution and thus vouch for its accuracy. It takes effect immediately and I have my first case to hear from where sloughing followed its administration.—Summary.

MATRIX FOR JACKET-CROWN.—To most dentists the making of a matrix for the jacket-crown is a much-dreaded proposition. My idea is to make this matrix of platinum wire which is a little heavier than the ordinary platinum wire used in the electric furnace. The idea of preparing the matrix for the crown is to wind the tooth with platinum wire in much the same way as a sailor would wind the end of a rope. This gives a cone-shaped matrix of platinum which is corrugated on the outside and on the inside as well. An impression is then taken in plaster or in modeling composition, pour up with any suitable investment material. The impression material is removed, leaving a spiral cone of platinum on the model, which can then be built up with porcelain and baked in the usual way. The model may be placed on the articulator and the piece broken off so that it can be tried into the mouth.—Dr. G. L. Hutchinson, Brief.

Annealing Gold.—Most operators are in the habit of heating their gold by passing it through the flame of a spirit lamp, or a Bunsen burner, but in either instance we are never certain of always having a pure flame; besides there is not one in a dozen that understands that he is removing gases and not developing cohesion, which is an inherant quality of the pure clean gold itself. To remove these gases uniformly and completely a comparatively low heat and more time is required and this is best accomplished with an electric annealer. It has been shown by experiment that the cohesive property of ordinary gold foil as it comes from the manufacturers shows itself at about 250 degrees F. and increases from this point to about 375 degrees F., after which nothing is gained. As this heat is not visible many dentists who have not tried the gold annealer with its low heat believe that gold annealed in this way is not thoroughly annealed, but clinical use shows it to be most cohesive.—Dr. M. L. Ward, Amer. D. Jour.

How to Improve Your Crown and Bridge Articulator.—The ordinary crown and bridge articulators now on the market have but one movement, namely, vertical. By the use of the mechanical saw and a few minutes of your spare time, enlarge the hole for the pin backwards say one-quarter of an inch, making a slit for the pin to slide back and forth. Do the same on both sides, but only in the one piece (lower), and fasten a small rubber band to each endof the pin, allowing it to pass around in front. This elastic band will keep the articulator in a given position when at rest, but can easily be moved to give any desired position, vertical, lateral and antero-posterior movements, which correspond to the movements of the jaws. This simple method will save the trouble and time of making the plaster articulator and is much better.—Dr. A. F. Donahower, Brief.

Treating an Alveolar Abscess.—I believe it is a mistake to carry a tooth along through weeks and weeks of treatment through the pulp chamber, and many of us make a mistake in treating teeth too often. Whenever an abscess forms about a root it nearly always surrounds the apex of the root. It destroys the peridental membrane around the sides of the root, consequently treatment through the root canal does not get at what we want, and after pus has been present for some time, if the surface of the root has been bathed in pus for many weeks, we will get satisfactory results from treatment through the pulp chamber in very few cases. I do not practice immediate root filling in such cases, but I have done it more immediately than is the general custom. In such cases I prefer to fill the root canal, and if necessary, make an opening through the alveolar process.—Dr. A. D. Black, Summary.

Drainage of Abscesses.—There is no place in the human body where care in securing beyond question the complete drainage of abscesses is so important as in the mucous tissues of the mouth. The blood supply is so rich in this region that cuts close in a surprisingly short time and the formation of pus is often profuse and rapid. Hence we must insist upon the necessity of keeping up the drainage for a sufficient time for the complete cleaning of the abscess. In all cases of the drainage of abscesses in the membranes of the mouth I insist on the use of 95 per cent. carbolic acid in the tent. A very small amount retained in the gauze or cotton is sufficient to cauterize the lips of the incision, making the opening freer, promoting the egress of pus and presenting a barrier to the ingress of new infective material.—Dr. G. V. Black, Northwestern Dental Journal.

